# Modern Sociology

**Published:** 1902-02-08

**Authors:** 


### Modern Sociology.

### SANITATION IN INDIA.

(*Concluded from page* 311.)

It is true that in Northern India caste prejudice is dying out to a certain extent; members of the lowest castes are not compelled, as they still are in some parts of the Madras Presidency, to leave the road when a Brahmin comes along lest their shadow should fall on him. But the impossibility of inducing any except men of low caste to undertake the work constantly hampers the sanitary service in India. The sanitary reformer is obliged to employ men who are regarded with the utmost prejudice, and to contend, as it were, with a horror of the whole matter. He is further hampered by the difficulty that permeates the whole of the Indian administration, the untrustworthiness and corruption of the native subordinates. Oppression tempered by bribery has been the native official's watchword for generations, and is unfortunately still. The venality of the police is a serious menace to the stability of British rule, and a new departure in any department opens the field to fresh abuses. For instance, there was lately a serious strike of cabmen and carters in Calcutta, and official inquiry has revealed the fact that it was largely due to the systematic blackmailing of the men, not only by the police, but by the agents of the Society for the Prevention of Cruelty to Animals. It must not be forgotten that Englishmen form a very small proportion of the officials of the Government of India, and though the standard of purity is slowly rising, yet, partly from inertia, partly from timidity, a native does not regard blackmailing and bribery as we do. It was largely for this reason that English soldiers were sent round with the search parties when plague first broke out in Poona, a course of action that was not very successful, largely on account of the lies published by certain sections of the native press.

These various difficulties might, however, be overcome if the zenana system did not lie at the very root of the whole social fabric of India. The native is, as a rule, a good tempered and much enduring individual, but he resents with the utmost bitterness anything which he believes may be an attack on the sanctity of his religion, or the invisibility of his women. And in the latter respect, except in a few isolated instances, there is no sign of change; in most parts of the country every man who can afford to do so still shuts up his womenkind from the eyes of all males but a few near relations. And they themselves, far from resenting this action, welcome it as a sign of delicacy and refinement, as a proof of affection for them and appreciation of their charms.

A native lady does not, as in Turkey, consider it enough to go about veiled; she never stirs from home except in a closely curtained chair; which if she goes a journey by train is brought to the door of the compartment and the curtains held over her as she steps in. So strict is the seclusion that it is considered a breach of good manners to even mention female relations, and their names need not be given in the census enumerations; an Englishwoman talking to a native gentleman would merely vaguely inquire, "Is your household well?" And so it follows that what goes on in the zenana is absolutely hidden from the outside world, and must be so as long as this seclusion is regarded as one of the fundamental principles of respectability. But while it lasts how can the necessary treatment of such a disease as plague be carried out in the face of ignorance and prejudice; the isolation, examination of all suspected persons, evacuation, and thorough disinfection of the premises? It might be done, perhaps, if the supply of lady doctors ard responsible English officials was sufficient. For the people have the most touching belief in the Englishman, and his good faith and straightforwardness. I have seen a Brahmin accused of harbouring stolen goods weeping at the door of the magistrate of the district imploring that the English superintendent of police might be sent to search his house and not a native official. But there are not enough of either officers or doctors, nor can there be enough without increasing taxation to an intolerable extent. And while the sanctity of the zenana is regarded with such feelings—feelings which have a force almost incredible to the European, it is easy to understand how necessary it is to take no step which may put undue power in the hands of possibly corrupt and venal underlings, or give any ground to the belief that the principle of seclusion is to be violated. For if such ideas were to spread, the whole country would rise in revolt, and the very foundations of government be shaken.

This may seem strong language to those to whom the maintenance of law and order represent on axiomatic condition of things inherited from their forefathers. But what was the state of the greater part of India a hundred years ago, of Oudh in the fifties, of Upper Burmah and other outlying tracts quite recently? The *pax Britannica* has brought security of life and property, but it can only exist by the passive acquiescence, if not the active help of the people. They recognise as they did in the days of the Mutiny, dimly enough it may be, that the present Government is on the whole a good one, and so they yield it obedience if not liking. It is this sentiment, and not the company of English soldiers a hundred miles away, that supports the civil officer, the only European it may be in a district of a million souls. But the bulk of the people are illiterate and very ignorant, prone to listen to ill-founded rumours, mistrustful of anything new, easily carried away when once their passions are aroused. And there are many individuals and classes besides the roughs in the great cities who would welcome the overthrow of the English Raj. Princes hemmed in by responsibilities and rules and regulations, remember that in their forefathers' time the ruler's will was law: ambitious soldiers of fortune hope to rise in the general confusion; the hereditary dacoits and poisoners, and thieves and cattle-stealers object to a government which has put an end to their occupations; Mahomedans and Brahmins alike trust that their religion would rise triumphant and all-conquering from the struggle; many regard as oppressive the suppression of suttee and female infanticide and slavery. That such representatives of disorder are only awaiting their opportunity is well known to Government, and proofs have not been wanting in recent riots, as at Cawnpore in 1900 when the plague hospital was wrecked and burnt down, and the policemen on duty killed, and their bodies flung into the flames. It was in consequence of this outbreak that no attempt was made in Benares a few months later to enforce compulsory notification and isolation when plague appeared there. But as long as the bulk of the people passively, if not openly, support the Government, the promoters of disorder can do little. Once, however, let this support be withdrawn, and who can tell what the end may be? In the opinion of many experienced and competent judges the attempt to enforce too hastily the principles of Western sanitation amongst races who do not understand it, and not only do not want it but violently object to it, may easily lead to a second and graver Mutiny.

